# Correlation of Air Pollution and Prevalence of Acute Pulmonary Embolism in Northern Thailand

**DOI:** 10.3390/ijerph191912808

**Published:** 2022-10-06

**Authors:** Chaiwat Bumroongkit, Chalerm Liwsrisakun, Athavudh Deesomchok, Chaicharn Pothirat, Theerakorn Theerakittikul, Atikun Limsukon, Konlawij Trongtrakul, Pattraporn Tajarernmuang, Nutchanok Niyatiwatchanchai, Juntima Euathrongchit, Juthamas Inchai, Warawut Chaiwong

**Affiliations:** 1 Division of Pulmonary, Critical Care, and Allergy, Department of Internal Medicine, Faculty of Medicine, Chiang Mai University, Chiang Mai 50200, Thailand; 2 Department of Radiology, Faculty of Medicine, Chiang Mai University, Chiang Mai 50200, Thailand

**Keywords:** air pollution, particular matter, acute pulmonary embolism, deep vein thrombosis, cardiopulmonary

## Abstract

Background: The relationship between the level of air pollution and acute pulmonary embolism (APE) has had inconsistent results. Objective: This study aimed to analyze the relationship between the high level of air pollution exposure and APE. Methods: A ten-year retrospective cohort, single-center study was performed on patients diagnosed with APE from October 2010 to December 2020. The association between air pollution and monthly APE case diagnosis was analyzed. Results: A total number of 696 patients was included. The effect of every 10 µg/m^3^ increment of particulate matters with an aerodynamic diameter < 10 µm (PM_10_) on total monthly APE cases (unprovoked PE and provoked PE) was increased significantly at lag 4, 5 and 6 months with adjusted RR (95% CI) of 1.06 (1.01, 1.12), *p* = 0.011, 1.07 (1.01, 1.13), *p* = 0.021 and 1.06 (1.01, 1.12), *p* = 0.030, respectively. Adjusted RR for APE was significantly increased for PM_10_ in the second tertile ((adjusted RR (95% CI) 1.76 (1.12, 2.77)), *p* = 0.014. Conclusions: We conclude that PM_10_ is associated with an increased prevalence of APE cases. The policy for tighter control of air pollution in our country is needed to reduce the impact of air pollutants on people’s health.

## 1. Introduction

The harmful effects of various ambient air pollution on human health, particularly in developing countries, have been persistently reported in many studies [1,2,3,4]. Both long-term and short-term exposures to ambient particulate matters (PMs), including PM with an aerodynamic diameter < 10 µm (PM_10_) and PM with an aerodynamic diameter < 2.5 µm (PM_2.5_), and gaseous pollution, including nitrogen dioxide (NO_2_), volatile organic compounds (VOCs), including benzene, carbon monoxide (CO), sulfur dioxide (SO_2_) and ozone (O_3_), increase the risk of respiratory admission, cardiovascular admission, cardiovascular mortality and all-cause mortality [5,6,7]. There was evidence of associations between air pollutants and cardiovascular problems, such as myocardial infarction, stroke, heart failure, and atrial fibrillation [8,9,10,11]. A meta-analysis of studies conducted in China to assess the mortality effects of short-term exposure to air pollution showed that each 10 µg/m^3^ increase in PM_2.5_ was associated with a 0.38% (95% CI; 0.31, 0.45) increase in total mortality, a 0.51% increase in respiratory mortality (95% CI; 0.30, 0.73) and a 0.44% (95% CI; 0.33, 0.54) increase in cardiovascular mortality [12]. In the respiratory aspect, air pollutants are the cause and aggravating factor of many respiratory diseases, such as chronic obstructive pulmonary disease (COPD), asthma and lung cancer [13]. Increased ambient O_3_, NO_2_, PM_2.5_ and SO_2_ levels were consistently associated with increased hospital admission for asthma and pneumonia in various studies conducted in Hong Kong and Taipei [14,15,16,17].

Venous thromboembolism (VTE), a condition with blood clots in the deep venous system, is composed of pulmonary embolism (PE), deep venous thrombosis (DVT) or both. DVT is defined as a disease with blood clots in the deep vein, most commonly in the lower extremities. Acute pulmonary embolism (APE) refers to a situation where the pulmonary artery contains blood clots that most likely slip from DVT. APE, as a complication of DVT, was the most severe form of VTE [18]. Although exposure to PM has been strongly linked to cardiovascular and respiratory disorders, the effect on VTE, including PE and DVT, is still uncertain [19].

In Chiang Mai Province, Northern Thailand, air pollutant levels usually exceed the safety threshold during January and April, which are winter and pre-summer times in Thailand. The source of air pollutant emissions in our area comes from forest fires and the burning of agricultural remains after harvest. Our previous study demonstrated the effect of increasing PM_10_ concentrations from seasonal smog on asthma and COPD exacerbations [20]. Interestingly, there is growing evidence of an association between VTE and air pollution in many areas of the world. Nevertheless, the relationship between the level of air pollution and VTE had inconsistent results [21,22,23,24,25,26]. Therefore, our study aimed to analyze the relationship between the level of air pollution exposure and acute pulmonary embolism (APE) in Chiang Mai, Northern Thailand.

## 2. Materials and Methods

### 2.1. Study Design and Population

A retrospective data collection was conducted. All Asian patients, aged ≥15 years, who were admitted to Chiang Mai University Hospital between October 2010 and December 2020 with ICD-10 coding I 26.0 (pulmonary embolism with acute cor pulmonale) and I 26.9 (pulmonary embolism without acute cor pulmonale) were recruited into the study. All patients live in the northern part of Thailand. All medical records were reviewed to confirm the diagnosis of APE.

Diagnosis of APE was made when the presence of one of the following radiologic criteria was confirmed by a radiologist.

1. Demonstration of thrombus in the pulmonary artery and its branches by computed tomography pulmonary angiography (CTPA).
2. Demonstration of thrombus in the pulmonary artery and its branches by CT chest with contrast.

Patients who had predisposing factors for PE including active cancers were defined as provoked PE. Those with no predisposing factors were defined as unprovoked PE.

A total of 1560 patient records was collected from the ICD-10 coding diagnosis between October 2010 and December 2020; 864 were excluded due to age under 15 years, non-Asian ethnicities, repeated cases and no CT confirmation of diagnosis. A total number of 696 patients was included in the study.

Patients’ data including demographics, risk factors, date of diagnosis, clinical data and underlying conditions were recorded. Only cases with the first diagnosis of APE were included for analysis. This study was conducted at Faculty of Medicine, Chiang Mai University, Chiang Mai, Thailand.

### 2.2. Air Pollution Data

The levels of air pollutants were measured at sampling stations of the Pollution Control Department, Ministry of National Resources and Environment which are located in municipal areas of the Muang District, Chiang Mai Province, Thailand. The analysis methods were the non-dispersive infrared detection for CO concentrations, the pararosaniline technique for SO_2_ concentrations, the chemiluminescence technique for NO_2_ and O_3_ concentrations and the gravimetric technique for PM_10_ and PM_2.5_ concentrations [27]. The data were reported as monthly average concentrations of pollutants. The PM_2.5_ levels were officially reported after July 2016 in Thailand. Therefore, the correlation between PM_2.5_ and APE was not analyzed. We use the average 24 h air quality guidelines level of WHO (45 µg/m^3^ of PM_10_) for reference to the monthly average PM_10_ in this study [28].

The study was approved by the Research Ethics Committee of the Faculty of Medicine, Chiang Mai University (Institutional Review Board (IRB) approval number: MED-2564-08294).

### 2.3. Statistical Analysis

Results for numerical values were expressed as means ± standard deviation (SD) or median, IQR (Interquartile range) and those for categorical data were expressed as absolute frequencies and percentages. The association between a monthly number of acute PE cases and PM_10_ concentrations was analyzed by the application of general linear models (GLMs) with Poisson distribution. Poisson models with log links are often called log-linear models and are used for frequency data. To determine the association between the effects of PM_10_ on acute PE admissions, Poisson regression was used for analysis after adjustment for SO_2_, NO_2_, CO and O_3_. To assess the lag structure between the concentration of PM_10_ level and the number of acute PE cases, we initially examined separate models for each lag from zero to seven months after the APE diagnosis. The lag time zero (lag 0) is the month of an increase of 10 µg/m^3^ of PM_10_. Finally, risk regression analysis is applied to the data to estimate risk ratios (RRs) with 95% confidence intervals (CIs) of the independent variables in the constructed model. For case-crossover design, to allow comparison across pollutants, the monthly PM_10_, CO, NO_2_, O_3_ and SO_2_ concentrations were divided into tertiles and frequencies of cases were compared using Fisher exact test. The risk of PE due to high levels of pollutants was evaluated by comparing tertiles of each pollutant level by using the first tertile as a control point and calculating the RR and the 95% CI. All tests for statistical significance were two tailed and *p*-values below 0.05 were considered statistically significant. Statistical analysis was performed using a software package (StataCorp, College Station, TX, USA).

## 3. Results

A total number of 696 patients was included in the study. The patients’ characteristics and risk factors are demonstrated in Table 1. Most of them (58.9%) were female. The mean age was 57.7 ± 15.7 years old (min = 15, max = 98). Clinical suspicion of APE before CT confirmation was made in 468 of 696 patients (67.2%), while 228 patients (32.8%) were incidental PE. Provoked PE was found in 560 (80.5%) and unprovoked PE was found in 136 (19.5%). Unprovoked and provoked PE data are shown in Table 2.

The levels of PM_10_, SO_2_, NO_2_, CO and O_3_ were 45.4 ± 27.5 (17.2–132) µg/m^3^, 0.9 ± 0.6 (0.0–3.0) ppb, 9.6 ± 4.5 (1.0–23.0) ppb, 0.5 ± 0.2 (0.1–1.0) ppm and 24.3 ± 9.9 (6.0–47.0) ppb, respectively (Table 3). During the study period, there were 46 months of low PM_10_ (monthly average < 45 µg/m^3^) and 77 months of high PM_10_ (monthly average ≥ 45 µg/m^3^). The PM_10_ concentrations in low PM_10_ months and high PM_10_ months were 28.8 ± 7.4 and 72.5 ± 25.5 µg/m^3^, respectively (Table 4). The concentrations of other pollutants were also significantly higher in high-PM_10_ months.

**Figure 1:**
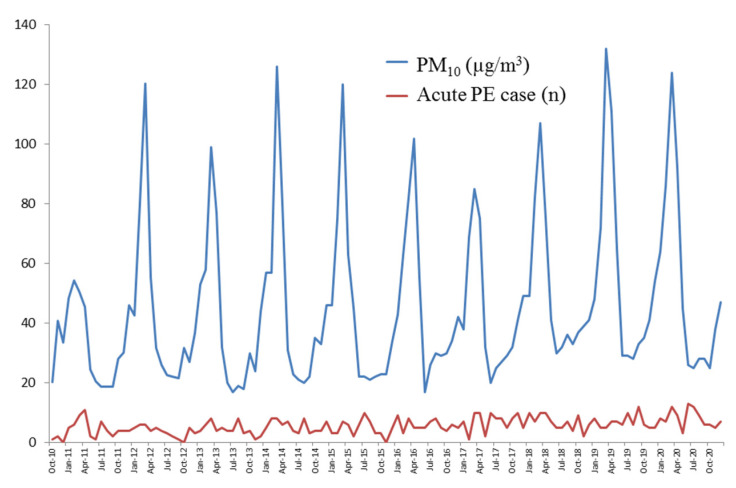
Variation in monthly average PM_10_ and monthly acute PE cases throughout the study period (October 2010–December 2020).

The monthly average of total APE cases, presented as median (IQR), was significantly higher in high-PM_10_ months (6 (5, 8)) than in low-PM_10_ months (5 (3, 7)), *p* = 0.013. The monthly average of unprovoked PE and provoked PE cases was insignificantly higher in high-PM_10_ months (Table 5). The effect of every 10 µg/m^3^ increase in PM_10_ on monthly total APE cases, unprovoked PE and provoked PE is demonstrated in Table 6. The total monthly APE cases increased significantly at lag 4, 5 and 6 months with adjusted RR (95% CI) of 1.06 (1.01, 1.12), *p* = 0.011; 1.07 (1.01, 1.13), *p* = 0.021 and 1.06 (1.01, 1.12), *p* = 0.030, respectively. Provoked PE cases increased significantly at lag 3, 4, 5 and 6 months with adjusted RR (95% CI) of 1.04 (1.01, 1.07), *p* = 0.004; 1.06 (1.03, 1.09), *p* < 0.001; 1.04 (1.01, 1.07), *p* = 0.004 and 1.04 (1.01, 1.07), *p* = 0.004, respectively. The risk of unprovoked PE was also increased but not statistically significant. Variation in monthly average PM_10_ and monthly acute PE cases throughout the study period are shown in Figure 1.

The tertiles of the air pollutant levels and the number of confirmed APE cases during the study period are shown in Table 7. There was a higher prevalence of APE cases in the second and third tertiles of PM_10_, SO_2_, CO and O_3_ than in the first tertile but not in the NO_2_ pollutant data. The relative risk for APE associated with exposure to elevated air pollutants is demonstrated in Table 8. When using the first tertile of air pollutants as a reference, the adjusted RR for APE was significantly increased for PM_10_ in the second (adjusted RR (95% CI) 1.76 (1.12, 2.77), *p* = 0.014) but not for NO_2_, CO and O_3_. For SO_2_, we could not divide data into tertiles; therefore, we used the cut-off median value as the lower range (0.0–0.9 ppb) and the higher range (≥1.0 ppb) for comparison. The higher-range SO_2_ had a non-significant increase in APE risk (adjusted RR (95% CI) 1.28 (0.85, 1.94), *p* = 0.236).

## 4. Discussion

Our study found that exposure to high concentrations of PM_10_ was significantly correlated with the number of confirmed APE cases. We used the reference level of PM_10_ as WHO air quality guideline recommendations [28]. In high-PM_10_ months (≥45 µg/m^3^), a monthly average of total pulmonary embolism cases was higher than low-PM_10_ (<45 µg/m^3^) months, *p* = 0.013. The results of our study are in agreement with those in previous studies, which showed an association between high concentrations of air pollutants and VTE incidence [29,30]. Baccarelli et al. proposed that the mechanism of PM-induced DVT triggered the pro-thrombotic state [25,31]. Although the numbers of unprovoked PE in our study did not meet statistical significance in high-PM_10_ months, the tendency of higher cases in those months might suggest the role of PM as a provoking factor in the unprovoked PE group. The trend was also observed in the number of provoked PE in high-PM_10_ months.

In Northern Thailand, illegal forest invasion for the cultivation of maize has been practiced for more than ten years [32]. Mass burning of agricultural waste preparing for the next cropping, joining together with forest firing in the dry season during January and April, which were winter and summer seasons in Thailand, has occurred since 2006. After that, air pollution became a major problem in Chiang Mai annually [32] for more than 15 years. At present, the problem is worse than ever, especially between March and April every year. Miguel-Díez et al. found that the hospitalization rate for PE was high in association with the autumn and winter seasons, lower temperatures and high levels of NO_2_ and O_3_ [33]. In contrast, Nimako et al. reported that the higher temperature was correlated with the high incidence of idiopathic PE [34]. However, the climate and temperature in Thailand are not as varied as in Western countries. We did not consider that climate change alone was the most important associated factor. A systematic review and meta-analysis concluded that the VTE rate was significantly high during winter time when it was usually coincident with increased PM in the same geographical areas [35].

To find the associations between the effect of an increase of every 10 µg/m^3^ of PM_10_ and total monthly acute PE cases, unprovoked PE and provoked PE, Poisson regression was used for analysis after adjustment for SO_2_, NO_2_, CO and O_3_. To assess the lag structure between the concentration of PM_10_ level and monthly PE cases, we initially examined separate models for each lag from lag time zero (lag 0), which is the month of PM_10_ measurement, to the lag later months, until no statistical significance was demonstrated. From our study, we found an association between every 10 µg/m^3^ increase in PM_10_ and increased risk of total APE cases after 4 months, 5 months and 6 months. The provoked PE risks also increased after 3, 4, 5 and 6 months. For unprovoked PE, the adjusted RR was also increased but without statistical significance. These findings are in agreement with the results in the study of Baccarelli et al., who found that exposure to a higher annual average PM_10_ level in the previous year was associated with shortened prothrombin time in DVT cases and every increment in 10 μg/m^3^ of PM_10_ increased DVT risk for 70% [25]. We supposed that the pro-thrombotic enhancement effect of PM to cause PE might take time after exposure.

Our study correlated the tertile of the air pollutant level and several confirmed APE cases and demonstrated a higher prevalence of APE cases in the second and third tertile of PM_10_, CO and O_3_ than in the first tertile. However, we did not find that NO_2_ and SO_2_ were significantly related to APE. These results were different from the study of Miguel-Diez et al. [26], which showed a relationship between short-term exposure to NO_2_ and the development of unprovoked PE. When using the first tertile of air pollutants as a reference, the adjusted RR of APE in the second and the third tertile was increased for PM_10_ but not for SO_2_, NO_2_, CO and O_3_. However, Gwon et al. [36] demonstrated that long-term exposures not only to PM_10_ but also to SO_2_ and O_3_ were correlated with the occurrence of VTE. However, not all studies replicated the same positive findings, making the association between exposure to air pollution and VTE development somewhat uncertain. Colais et al. found a strong relationship between short-term NO_2_ exposure and the development of PE, but not with PM_10_ and O_3_ [37]. A large prospective study conducted in the U.S. provided no association between short-term or long-term traffic-related air pollution exposure and the risk of VTE [38]. A study in Beijing by Li et al. showed that exposure to PM_2.5_, PM_10_, SO_2_ and CO increased PE admission [39]. A recent meta-analysis by Miao et al. demonstrated that exposure to CO, SO_2_, PM_10_ and PM_2.5_ had no significant association with PE. However, NO_2_ and O_3_ modestly increased the risk of PE [40]. Although the exact pathogenesis of air pollutants to VTE development was not well known, the possible hypotheses were pro-inflammatory effects and oxidative stress on alveolar epithelial cells and macrophages [41], endothelial malfunction [42], activation of the coagulation pathways [43,44,45,46,47], platelet aggregation [48] and in situ thrombus formation [49]. In addition, multifactorial and unknown factors, such as genetic heterogeneity risk for VTE [50], might be involved in the occurrence of pollution-associated PE and made the results of this relationship inconsistent.

Our study had some strengths. Firstly, we enrolled only Asian patients. Therefore, the heterogeneity in the study population might be minimal. In addition, our study was less affected by other confounding factors, including national policy or the effects of traffic-associated air pollution. Although the policy on reducing air pollution has been implemented by the Thai government since 1992, the air pollution remains a serious problem in the northern region of Thailand between January and April of every year. Moreover, the air pollution caused by traffic was not a major problem in the northern part of Thailand, including Chiang Mai, where most of these regions are rural areas [32]. Therefore, the temporal changes in air pollution and traffic regulations may not be affected by the results of this study. Secondly, our study is a large retrospective cohort. Thirdly, our air pollutant data were recorded completely for ten years for analysis of both short-term and long-term exposure effects of air pollutants. However, there are some limitations to this study. Firstly, the number of pollutants analyzed was limited, so we could not obtain complete data on PM_2.5_, which might be a better representative of a thrombosis-associated pollutant than PM_10_. Secondly, we did not evaluate the prevalence of DVT. Therefore, the rate of overall VTE induced by pollution was not known. Thirdly, the meteorological parameters, including temperature, humidity and wind speed, were not included as confounding factors for the analysis. Fourthly, occupational status was not included for the analysis. Therefore, this should be counted as a confounding factor in a future study.

## 5. Conclusions

Our study shows a significant association between a high level of PM_10_ and an increased prevalence of APE cases. This study provides evidence in support of an association between exposure to particulate air pollution and the risk of VTE. The policy for higher control of air pollution in our country is needed to reduce its impact on our citizens’ health.

## Figures and Tables

**Table 1:** Demographic data, clinical and underlying conditions.

Demographic Data (n = 696)	Mean ± SD or n (%)
Age (years) (Range)	57.7 ± 15.7 (15–98)
Male sex	286 (41.1)
Female sex	410 (58.9)
Clinical type	
Suspected PE	468 (67.2)
Incidental PE	228 (32.8)
Provoked PE	560 (80.5)
Unprovoked PE	136 (19.5)
Underlying conditions	
Hypertension	305 (43.8)
DM	114 (16.4)
Renal diseases	69 (9.9)
Thalassemia and hematologic diseases	45 (6.5)
COPD	38 (5.5)
Cirrhosis	31 (4.5)
CAD with prior myocardial infarction	30 (4.3)
Other chronic lung problem	28 (4.0)
Active smoking	24 (3.4)
Chronic alcohol drinking	24 (3.4)
Connective tissue disease	22 (3.2)
OSA	15 (2.2)
Obesity (BMI ≥ 30 kg/m^2^)	12 (1.7)
Nephrotic syndrome	11 (1.6)
Post-splenectomy	10 (1.4)
HIV	9 (1.3)
Vasculitis	3 (0.4)
Pregnancy	2 (0.3)
Known prothrombotic state	68 (9.8)
Known prothrombotic state (N = 68)	
Protein C deficiency	31 (45.6)
Protein S deficiency	12 (17.6)
AT III deficiency	6 (8.8)
lupus anticoagulant	15 (22.1)
Anticardiolipin	2 (2.9)

Note: Data are mean ± standard deviation (SD) or n (%). Abbreviations: AT, antithrombin-III; DM, diabetes mellitus; COPD, chronic obstructive pulmonary disease; CAD, coronary arterial disease; OSA, obstructive sleep apnea; HIV, human immunodeficiency viral infection; PE, pulmonary embolism.

**Table 2:** Provoked and unprovoked PE.

Characteristics	n (%)
Unprovoked	136 (19.5)
Provoked PE	560 (80.5)
Active malignancy	388 (55.7)
Immobility—total body immobilization	211 (30.4)
Surgery or trauma requiring endotracheal or epidural anesthesia within the last 4 weeks	141 (20.3)
Indwelling venous catheter	23 (3.3)
Recent significant trauma	12 (1.7)
Oral contraceptives/Estrogen therapy	27 (3.9)
Protein C deficiency	31 (4.4)
Protein S deficiency	12 (1.7)
AT III deficiency	6 (0.9)
lupus anticoagulant	15 (2.1)
Antiphospholipid	2 (0.3)
Right side endocarditis	2 (0.3)
Long travel history > 6 h	10 (1.4)

Note: Data are n (%). Abbreviations: AT, antithrombin-III; PE, pulmonary embolism.

**Table 3:** Monthly pollutant data (October 2010 to December 2020).

Pollutants	Mean ± SD	Min–Max
PM_10_ (µg/m^3^)	45.4 ± 27.5	17.0–132
SO_2_ (ppb)	0.9 ± 0.6	0.0–3.0
NO_2_ (ppb)	9.6 ± 4.5	1.0–23.0
CO (ppm)	0.5 ± 0.2	0.1–1.0
O_3_ (ppb)	24.3 ± 9.9	6.0–47.0

Note: Data are mean ± standard deviation (SD). Abbreviations: PM_10_, particulate matters with diameter of less than 10 microns; CO, carbon monoxide; SO_2_, sulfur dioxide; NO_2_, nitrogen dioxide; O_3_, ozone.

**Table 4:** Pollutant data between high-PM_10_ period (≥45 µg/m^3^) and low-PM_10_ periods (<45 µg/m^3^) recommended by WHO air quality guidelines 2021.

Pollutants	Low PM_10_ (n = 46)	High PM_10_ (n = 77)	*p*-Value
PM_10_ (µg/m^3^)	28.8 ± 7.4	72.5 ± 25.5	<0.001
SO_2_ (ppb)	0.8 ± 0.6	1.0 ± 0.8	0.031
NO_2_ (ppb)	7.2 ± 2.3	13.5 ± 4.7	<0.001
CO (ppm)	0.4 ± 0.1	0.6 ± 0.2	<0.001
O_3_ (ppb)	18.6 ± 6.0	33.7 ± 7.4	<0.001

Note: Data are mean ± standard deviation (SD). Abbreviations: PM_10_, particulate matters with diameter of less than 10 microns; CO, carbon monoxide; SO_2_, sulfur dioxide; NO_2_, nitrogen dioxide; O_3_, ozone.

**Table 5:** Acute pulmonary emboli case during high-PM_10_ months (≥45 µg/m^3^) and low-PM_10_ months (<45 µg/m^3^).

Variables	Low PM_10_	High PM_10_	*p*-Value
Monthly average pulmonary emboli case	5.0 (3.0, 7.0)	6.0 (5.0, 8.0)	0.013
Monthly average unprovoked pulmonary emboli case	1.0 (0.0, 2.0)	1.0 (1.0, 2.0)	0.111
Monthly average provoked pulmonary emboli case	4.0 (2.0, 6.0)	4.5 (3.0, 6.0)	0.678

Notes: Results are expressed as median (IQR).

**Table 6:** Associations between every 10 µg/m^3^ increase in PM_10_ and monthly APE cases (total), unprovoked PE and provoked PE (October 2010–December 2020).

Outcomes	Adjusted RR ^#^ (95% CI)	*p*-Value
Total acute PE cases		
Lag 0 month	1.00 (0.92, 1.10)	0.865
Lag 1 month	1.00 (0.95, 1.06)	0.919
Lag 2 month	1.02 (0.98, 1.06)	0.402
Lag 3 month	1.02 (0.98, 1.06)	0.275
Lag 4 month	1.06 (1.01, 1.12)	0.011
Lag 5 month	1.07 (1.01, 1.13)	0.021
Lag 6 month	1.06 (1.01, 1.12)	0.030
Lag 7 month	1.01 (0.98, 1.04)	0.550
Unprovoked PE		
Lag 0 month	1.00 (0.94, 1.07)	0.887
Lag 1 month	1.03 (0.97, 1.09)	0.361
Lag 2 month	1.03 (0.97, 1.09)	0.280
Lag 3 month	1.05 (0.99, 1.10)	0.109
Lag 4 month	1.05 (0.99, 1.11)	0.111
Lag 5 month	1.03 (0.97, 1.09)	0.310
Lag 6 month	1.03 (0.97, 1.09)	0.328
Lag 7 month	1.02 (0.96, 1.09)	0.445
Provoked PE		
Lag 0 month	1.02 (0.98, 1.05)	0.326
Lag 1 month	1.02 (0.99, 1.04)	0.237
Lag 2 month	1.03 (0.99, 1.06)	0.091
Lag 3 month	1.04 (1.01, 1.07)	0.004
Lag 4 month	1.06 (1.03, 1.09)	<0.001
Lag 5 month	1.04 (1.01, 1.07)	0.004
Lag 6 month	1.04 (1.01, 1.07)	0.004
Lag 7 month	1.00 (0.97, 1.03)	0.773

Note: RR, relative risk; ^#^, adjusted for other pollutants (CO, SO_2_, NO_2_ and O_3_). Abbreviations: PM_10_, particulate matters with diameter of less than 10 microns; RR, relative risk; PE, pulmonary embolism.

**Table 7:** Tertiles of exposure to air pollutants of study population during months of enrollment.

Pollutants	Tertiles	No. of Cases	*p*-Value
PM_10_ (μg/m^3^)	≤26.0	116	0.045
	26.1–54.9	349	
	≥55.0	181	
SO_2_ (ppb)	0.0–0.9	209	0.366
	≥1.0	487	
NO_2_ (ppb)	≤7.9	341	0.349
	8.0–11.9	179	
	≥12.0	176	
CO (ppm)	≤0.32	86	0.053
	0.33–0.59	130	
	≥0.60	87	
O_3_ (ppb)	≤15.0	161	0.471
	15.1–38.9	341	
	≥39.0	194	

Abbreviations: PM_10_, particulate matters with diameter of less than 10 microns; CO, carbon monoxide; SO_2_, sulfur dioxide; NO_2_, nitrogen dioxide; O_3_, ozone.

**Table 8:** Relative risk for APE associated with exposure to elevated air pollutants.

Pollutants	Tertiles	Unadjusted RR (95% CI)	*p*-Value	Adjusted RR ^#^ (95% CI)	*p*-Value
PM_10_ (μg/m^3^)	≤26.0	Ref.		Ref.	
	26.1–54.9	1.12 (0.93, 1.35)	0.225	1.76 (1.12, 2.77)	0.014
	≥55.0	1.13 (0.91, 1.39)	0.271	1.62 (0.90, 3.05)	0.105
SO_2_ (ppb) *	0.0–0.9	Ref.		Ref.	
	≥1.0	0.85 (0.73, 1.00)	0.057	1.28 (0.85, 1.94)	0.236
NO_2_ (ppb)	≤7.9	Ref.		Ref.	
	8.0–11.9	0.72 (0.60, 0.87)	<0.001	0.75 (0.51, 1.09)	0.140
	≥12.0	0.75 (0.63, 0.90)	0.002	0.83 (0.48, 1.45)	0.518
CO (ppm)	≤0.32	Ref.		Ref.	
	0.33–0.59	0.82 (0.63, 1.08)	0.165	0.73 (0.53, 1.02)	0.068
	≥0.60	1.01 (0.75, 1.36)	0.939	0.76 (0.48, 1.20)	0.243
O_3_ (ppb)	≤15	Ref.		Ref.	
	15.1–38.9	0.96 (0.79, 1.15)	0.642	0.76 (0.52, 1.11)	0.163
	≥39.0	1.02 (0.83, 1.26)	0.835	0.92 (0.56, 1.49)	0.728

Note: * SO_2_ data could not be divided into tertiles; we used cut-off median value as lower range and higher range. Abbreviations: PM_10_, particulate matters with diameter of less than 10 microns; CO, carbon monoxide; SO_2_, sulfur dioxide; NO_2_, nitrogen dioxide; O_3_, ozone; ^#^ adjusted for all pollutants (PM_10_, CO, SO_2_, NO_2_ and O_3_).

## Data Availability

The data that support the findings of this study are available on request from the corresponding author.

## References

[1] Lim S.S., Vos T., Flaxman A.D., Danaei G., Shibuya K., Adair-Rohani H., Amann M., Anderson H.R., Andrews K.G., Aryee M. (2012). A comparative risk assessment of burden of disease and injury attributable to 67 risk factors and risk factor clusters in 21 regions, 1990–2010: A systematic analysis for the Global Burden of Disease Study 2010. Lancet.

[2] Mannucci P.M., Harari S., Martinelli I., Franchini M. (2015). Effects on health of air pollution: A narrative review. Intern. Emerg. Med..

[3] Landrigan P.J. (2017). Air pollution and health. Lancet Public Health.

[4] Mannucci P.M., Franchini M. (2017). Health effects of ambient air pollution in developing countries. Int. J. Environ. Res. Public Health.

[5] Dominici F., Peng R.D., Bell M.L., Pham L., McDermott A., Zeger S.L., Samet J.M. (2006). Fine particulate air pollution and hospital admission for cardiovascular and respiratory diseases. J. Am. Med. Assoc..

[6] Raaschou-Nielsen O., Andersen Z.J., Jensen S.S., Ketzel M., Sørensen M., Hansen J., Loft S., Tjønneland A., Overvad K. (2012). Traffic air pollution and mortality from cardiovascular disease and all causes: A Danish cohort study. Environ. Health.

[7] Samoli E., Atkinson R.W., Analitis A., Fuller G.W., Green D.C., Mudway I., Anderson H.R., Kelly F.J. (2016). Associations of Short-term Exposure to Traffic-related Air Pollution with Cardiovascular and Respiratory Hospital Admissions in London, UK. Occup. Environ. Med..

[8] Rajagopalan S., Al-Kindi S.G., Brook R.D. (2018). Air Pollution and Cardiovascular Disease: JACC State-of-the-Art Review. J. Am. Coll. Cardiol..

[9] Scheers H., Jacobs L., Casas L., Nemery B., Nawrot T.S. (2015). Long-term exposure to particulate matter air pollution is a risk factor for stroke. Stroke.

[10] Von Klot S., Peters A., Aalto P., Bellander T., Berglind N., Ippoliti D.D., Elosua R., Hörmann A., Kulmala M., Lanki T. (2005). Ambient air pollution is associated with increased risk of hospital cardiac readmissions of myocardial infarction survivors in five European cities. Circulation.

[11] Solimini A.G., Renzi M. (2017). Association between air pollution and emergency room visits for atrial fibrillation. Int. J. Environ. Res. Public Health.

[12] Shang Y., Sun Z., Cao J., Wang X., Zhong L., Bi X., Li H., Liu W., Zhu T., Huang W. (2013). Systematic review of Chinese studies of short-term exposure to air pollution and daily mortality. Environ. Int..

[13] Jiang X.Q., Mei X.D., Feng D. (2016). Air pollution and chronic airway diseases: What should people know and do?. J. Thorac. Dis..

[14] Qiu H., Yu I.T., Tian L., Wang X., Tse L.A., Tam W., Wong T.W. (2012). Effects of coarse particulate matter on emergency hospital admissions for respiratory diseases: A time-series analysis in Hong Kong. Environ. Health Perspect..

[15] Ko F.W., Tam W., Wong T.W., Lai C.K., Wong G.W., Leung T.F., Ng S.S., Hui D.S. (2007). Effects of air pollution on asthma hospitalization rates in different age groups in Hong Kong. Clin. Exp. Allergy.

[16] Cheng M.H., Chen C.C., Chiu H.F., Yang C.Y. (2014). Fine particulate air pollution and hospital admissions for asthma: A case-crossover study in Taipei. J. Toxicol. Environ. Health.

[17] Qiu H., Tian L.W., Pun V.C., Ho K.F., Wong T.W., Yu I.T. (2014). Coarse particulate matter associated with increased risk of emergency hospital admissions for pneumonia in Hong Kong. Thorax.

[18] Cordeanu E.M., Lambach H., Heitz M., Di Cesare J., Mirea C., Faller A.M., Cavaro A.C., Frantz A.S., Gaertner S., Schini-Kerth V. (2019). Pulmonary Embolism and Coexisting Deep Vein Thrombosis: A Detrimental Association?. J. Clin. Med..

[19] Franchini M., Mengoli C., Cruciani M., Bonfanti M., Mannucci P.M. (2016). Association between particulate air pollution and venous thromboembolism: A systematic literature review. Eur. J. Intern. Med..

[20] Pothirat C., Tosukhowong A., Chaiwong W., Liwsrisakun C., Inchai J. (2016). Effects of seasonal smog on asthma and COPD exacerbations requiring emergency visits in Chiang Mai, Thailand. Asian Pac. J. Allergy Immunol..

[21] Franchini M., Guida A., Tufano A., Coppola A. (2012). Air pollution, vascular disease and thrombosis: Linking clinical data and pathogenic mechanisms. J. Thromb. Haemost..

[22] Crous-Bou M., Harrington L.B., Kabrhel C. (2016). Environmental and genetic risk factors associated with venous thromboembolism. Semin. Thromb. Hemost..

[23] Dales R.E., Cakmak S., Vidal C.B. (2010). Air pollution and hospitalization for venous thromboembolic disease in Chile. J. Thromb. Haemost..

[24] Spiezia L., Campello E., Bon M., Maggiolo S., Pelizzaro E., Simioni P. (2014). Short-term exposure to high levels of air pollution as a risk factor for acute isolated pulmonary embolism. Thromb. Res..

[25] Baccarelli A., Martinelli I., Zanobetti A., Grillo P., Hou L.F., Bertazzi P.A., Mannucci P.M., Schwartz J. (2008). Exposure to particulate air pollution and risk of deep vein thrombosis. Arch. Intern. Med..

[26] de Miguel-Diez J., Blasco-Esquivias I., Rodriguez-Matute C., Bedate-Diaz P., Lopez-Reyes R., Fernandez-Capitan C., Garcia-Fuika S., Lobo-Beristain J.L., Garcia-Lozaga A., Quezada C.A. (2020). Correlation between short-term air pollution exposure and unprovoked lung embolism. Prospective observational (Contamina-TEP Group). Thromb. Res..

[27] Air Quality and Noise Management Bureau, 2004. http://www.pcd.go.th/info.

[28] WHO Global Air Quality Guidelines: Particulate Matter (PM_2.5_ and PM_10_), Ozone, Nitrogen Dioxide, Sulfur Dioxide and Carbon Monoxide. https://apps.who.int/iris/handle/10665/345329.

[29] Martinelli N., Girelli D., Cigolini D., Sandri M., Ricci G., Rocca G., Olivieri O. (2012). Access rate to the emergency department for venous thromboembolism in relationship with coarse and fine particulate matter air pollution. PLoS ONE.

[30] Chiu H.H., Whittaker P. (2013). Venous thromboembolism in an industrial north American city: Temporal distribution and association with particulate matter air pollution. PLoS ONE.

[31] Baccarelli A., Martinelli I., Pegoraro V., Melly S., Grillo P., Zanobetti A., Hou L., Bertazzi P.A., Mannucci P.M., Schwartz J. (2009). Living near major traffic roads and risk of deep vein thrombosis. Circulation.

[32] Pengchai P., Chantara S., Sopajaree K., Wangkarn S., Tengcharoenkul U., Rayanakorn M. (2009). Seasonal variation, risk assessment and source estimation of PM 10 and PM10-bound PAHs in the ambient air of Chiang Mai and Lamphun, Thailand. Environ. Monit. Assess..

[33] de Miguel-Díez J., Jiménez-García R., López de Andrés A., Hernández-Barrera V., Carrasco-Garrido P., Monreal M., Jiménez D., Jara-Palomares L., Álvaro-Meca A. (2016). Analysis of environmental risk factors for pulmonary embolism: A case-crossover study (2001–2013). Eur. J. Intern. Med..

[34] Nimako K., Poloniecki J., Draper A., Rahman T. (2012). Seasonal variability and meteorological factors: Retrospective study of the incidence of pulmonary embolism from a large United Kingdom teaching hospital. Respir. Care.

[35] Dentali F., Ageno W., Rancan E., Donati A.V., Galli L., Squizzato A., Venco A., Mannucci P.M., Manfredini R. (2011). Seasonal and monthly variability in the incidence of venous thromboembolism. A systematic review and a meta-analysis of the literature. Thromb. Haemost..

[36] Gwon J.G., Lee S.A., Park K.Y., Oh S.U., Kim J.S., Seo H.M. (2022). Long-Term Exposure to Air Pollution and Incidence of Venous Thromboembolism in the General Population: A Population-Based Retrospective Cohort Study. J. Clin. Med..

[37] Colais P., Serinelli M., Faustini A., Stafoggia M., Randi G., Tessari R., Chiusolo M., Pacelli B., Mallone S., Vigotti M.A. (2009). Air pollution and urgent hospital admissions in nine Italian cities. Epidemiol. Prev..

[38] Kan H., Folsom A.R., Cushman M., Rose K.M., Rosamond W.D., Liao D., Lurmann F., London S.J. (2011). Traffic exposure and incident venous thromboembolism in the Atherosclerosis Risk in Communities (ARIC) Study. J. Thromb. Haemost..

[39] Li Z., Zhang Y., Yuan Y., Yan J., Mei Y., Liu X., Xu Q., Shi J. (2022). Association between exposure to air pollutants and the risk of hospitalization for pulmonary embolism in Beijing, China: A case-crossover design using a distributed lag nonlinear model. Environ. Res..

[40] Miao H., Li X., Wang X., Nie S. (2021). Air pollution increases the risk of pulmonary embolism: A meta-analysis. Rev. Environ. Health.

[41] Li X.Y., Gilmour P.S., Donaldson K., MacNee W. (1996). Free radical activity and proinflammatory effects of particulate air pollution (PM_10_) in vivo and in vitro. Thorax.

[42] Mills N.L., Donaldson K., Hadoke P.W., Boon N.A., MacNee W., Cassee F.R., Sandström T., Blomberg A., Newby D.E. (2009). Adverse cardiovascular effects of air pollution. Nat. Clin. Pract. Cardiovasc. Med..

[43] Baccarelli A., Zanobetti A., Martinelli I., Grillo P., Hou L., Giacomini S., Bonzini M., Lanzani G., Mannucci P.M., Bertazzi P.A. (2007). Effects of exposure to air pollution on blood coagulation. J. Thromb. Haemost..

[44] Budinger G.R., McKell J.L., Urich D., Foiles N., Weiss I., Chiarella S.E., Gonzalez A., Soberanes S., Ghio A.J., Nigdelioglu R. (2011). Particulate matter-induced lung inflammation increases systemic levels of PAI-1 and activates coagulation through distinct mechanisms. PLoS ONE.

[45] Emmerechts J., Jacobs L., Van Kerckhoven S., Loyen S., Mathieu C., Fierens F., Nemery B., Nawrot T.S., Hoylaerts M.F. (2012). Air pollution-associated procoagulant changes: The role of circulating microvesicles. J. Thromb. Haemost..

[46] Franchini M., Mannucci P.M. (2011). Thrombogenicity and cardiovascular effects of ambient air pollution. Blood.

[47] Milojevic A., Wilkinson P., Armstrong B., Bhaskaran K., Smeeth L., Hajat S. (2014). Short-term effects of air pollution on a range of cardiovascular events in England and Wales: Case-crossover analysis of the MINAP database, hospital admissions and mortality. Heart.

[48] Rudez G., Janssen N.A., Kilinc E., Leebeek F.W., Gerlofs-Nijland M.E., Spronk H.M., ten Cate H., Cassee F.R., de Maat M.P. (2009). Effects of ambient air pollution on hemostasis and inflammation. Environ. Health Perspect..

[49] Nemmar A., Hoylaerts M.F., Hoet P.H., Dinsdale D., Smith T., Xu H., Vermylen J., Nemery B. (2002). Ultrafine particles affect experimental thrombosis in an in vivo hamster model. Am. J. Respir. Crit. Care Med..

[50] Angchaisuksiri P., Atichartakarn V., Aryurachai K., Archararit N., Rachakom B., Atamasirikul K., Tiraganjana A. (2007). Risk factors of venous thromboembolism in Thai patients. Int. J. Hematol..

